# ^18^F-FDG Brain PET/CT Metabolic Imaging in Patients with Suspected Autoimmune Encephalitis (AE) in the Early Stage: Can the Procedure Play a Complementary Diagnostic Role?

**DOI:** 10.3390/brainsci15020113

**Published:** 2025-01-24

**Authors:** Andrea Marongiu, Paolo Galleri, Antonio Mura, Paolo Solla, Giuseppe Madeddu, Angela Spanu, Susanna Nuvoli

**Affiliations:** 1 Unit of Nuclear Medicine, Department of Medicine, Surgery and Pharmacy, University of Sassari, 07100 Sassari, Italy; amarongiu2@uniss.it (A.M.); p.galleri1@studenti.uniss.it (P.G.); a.mura203@studenti.uniss.it (A.M.); giuseppe.madeddu@email.it (G.M.); aspanu@uniss.it (A.S.); 2 Unit of Neurology, Department of Medicine, Surgery and Pharmacy, University of Sassari, 07100 Sassari, Italy; psolla@uniss.it

**Keywords:** brain metabolism, autoimmune encephalitis, early stage, follow-up, ^18^F-FDG PET/CT, EEG, MRI, CSF

## Abstract

**Background/Objectives**: Recent studies reported that ^18^F-Fluorodeoxyglucose (FDG) positron –emission tomography/computed tomography (PET/CT), even in comparison with other traditional methods, can play a role in diagnosing AE and supporting early treatment. In the present study, we further investigated whether ^18^F-FDG PET/CT may be a complementary diagnostic tool to conventional procedures in patients with acute symptoms of suspected AE in the early phase. **Methods**: Eleven consecutive patients with recent acute symptoms suggestive of AE were retrospectively enrolled and underwent brain PET/CT after receiving an intravenous injection of 3.7 MBq/kg of ^18^F-FDG. **Results**: PET/CT showed abnormal FDG uptake in 9/11 patients classified as AE, while it was negative in the remaining 2/11 cases with vascular lesions. Magnetic resonance imaging (MRI), conducted in only 10/11 cases—one patient was a pacemaker wearer—identified suspected AE areas in 3/10 cases, ischemic lesions in another 3/10, and nonspecific data in the remaining 4/10 cases. Cerebrospinal fluid (CSF) tests revealed autoantibody delayed occurrence only in three patients (anti-GAD65, anti-Ma2, and anti-LGI1). After first-line treatment, 3/9 patients showed clinical improvement. Another 3/9 patients experienced partial improvement but with recurrence and new AE brain areas identified by PET/CT, which also detected favorable responses to second-line treatment in 2/3 cases. The remaining 3/9 patients, who were not responsive to treatment, ultimately died. **Conclusions**: In this study, PET/CT was effective in early identification of AE and enabling rapid therapy, even with inconclusive MRI and persistently negative or delayed positive CSF tests. PET/CT may aid in evaluating treatment response and detecting relapse. Notably, a negative PET/CT was associated with AE absence.

## 1. Introduction

Encephalitis is considered a severe brain inflammatory disease with a complex differential diagnosis and a poor prognosis, that can often lead to serious and irreversible consequences in up to 56% of survivors and even death in 7–18% of cases [1,2]. Historically, encephalitis has been thought to be primarily caused by infections. However, in recent years, an increase in the incidence and prevalence of encephalitis with an autoimmune origin, including its presence in paraneoplastic syndromes [3], has been observed compared to the infectious cause. Moreover, one rare but potential cause of acute disseminated encephalomyelitis (ADEM) complications could be linked to SARS-CoV-2 infection [4]. Additionally, the diagnosis of autoimmune encephalitis (AE) has improved over time [5,6,7,8]. The onset of AE can range from acute to subacute forms, and symptoms are varied and nonspecific. They can include memory deficits, behavior alterations, psychosis, seizures, movement disorders, autonomic symptoms, and coma [9]. In a large percentage of cases, AE is associated with the presence of autoantibodies against neuronal cell surface or intracellular antigens, including synaptic receptors or ion channels. The number of identified autoantibodies is progressively increasing [6,10], and the most frequent are those against the glioma-inactivated 1 protein (LGI-1), the N-methyl-D-aspartate receptor (NMDAR), and the glutamic acid decarboxylase 65-kDa isoform (GAD65) [11].

When other alternative diagnoses have been ruled out [12], the diagnosis of AE is based on clinical criteria [6], electroencephalogram (EEG), cerebrospinal fluid (CSF) examination, screening of serum tumor markers, infectious agents, and neuronal autoantibodies, and magnetic resonance imaging (MRI) [6].

However, despite the need to undertake adequate therapies as soon as possible to improve patients’ prognosis, scientific data obtained in several studies have shown that the clinical symptoms are heterogeneous and variable, especially in the early phase of the disease. Consequently, clinical criteria alone have shown low specificity [13,14,15].

Searching for specific autoantibodies in CSF and serum markers can be time-consuming. Initial CSF samples may test negative but later become positive or remain negative even when the disease is present. Additionally, autoantibody detection could yield irrelevant results or false positives [13,14,15].

The results of EEG are often nonspecific, and the use of standard MRI techniques such as T2 and Fluid-Attenuated Inversion Recovery (FLAIR) has shown low sensitivity and specificity. These techniques only show a hyperintense signal in a limited percentage of cases of AE [12,16,17].

In recent years, the use of brain ^18^F-fluorodeoxyglucose (FDG) positron emission tomography/computed tomography (PET/CT) has been emerging as a complementary and more sensitive diagnostic tool to the traditional methods used in AE and paraneoplastic syndromes, such as EEG, MRI, and CSF exams, obtaining valuable diagnostic performance [18,19,20].

Compared to these procedures, PET/CT is more frequently abnormal in the early phase of these conditions. In this retrospective study, conducted on a small group of patients with recent acute/subacute symptoms of suspected AE, the diagnostic complementary significance of PET/CT has been further explored in the early phase of AE, as well as in the follow-up of some affected patients. The study aimed to evaluate the potential complementary role of ^18^F-FDG PET/CT in the diagnostic protocol for encephalitis.

## 2. Materials and Methods

Eleven consecutive patients, labeled A to M, were retrospectively enrolled due to acute or subacute clinical symptoms suggestive of AE. Among these patients, nine were males and two were females, with ages ranging from 22 to 80 years (average age: 61.8 ± 18.1 years). Patient characteristics and clinical symptoms are illustrated in Table 1.

The inclusion criteria for suspected or possible AE were:

– Subacute onset of symptoms (rapid progression of less than 3 months) of working memory deficits (short-term memory loss), altered mental status, or psychiatric symptoms;
– Reasonable exclusion of alternative causes, with negative serum data for infectious/bacterial/viral agents of meningitis and infectious encephalitis;
– At least one of the following pieces of evidence: new focal CNS findings, seizures not explained by a previously known seizure disorder, supportive CSF, or MRI features suggestive of encephalitis [6,21].

However, seven other patients who did not fully satisfy the aforementioned criteria were excluded.

EEG results, MRI findings, serological and CSF data at the appearance of clinical symptoms are detailed in Table 2.

In particular, EEG tests were conducted in only nine cases due to poor cooperation in two patients, and results were either nonspecific or negative in all except two cases where seizures were present. In these two instances (cases C and L), an epileptic focus was identified. Serological tests, performed for all cases, showed negative results for tumor markers, bacterial or viral meningitis, and other infectious agents. An MRI was conducted on ten out of eleven patients, with the remaining case (case L) being a pacemaker wearer. In six of the ten cases, the MRI results indicated hyperintense areas on T2/FLAIR images. Specifically: three cases suggested AE (cases A, B, and D), two cases indicated ischemic lesions (cases E and F), and one case (case C) was deemed uncertain, potentially indicating either an ischemic lesion in the subacute phase, glial neoplasia, or encephalitis. In the remaining four cases (G, I, L, and M), the MRI results were nonspecific. Baseline CSF autoantibody tests were conducted for all cases, and these tests were repeated later if the initial results were negative. During the initial CSF tests, proteinorrachia was present in six cases (A, F, G, H, I, and M). Of these, two cases (A and G) showed bands of IgG and one case (M) exhibited pleocytosis together with proteinorrachia. In three cases (B, G, and L), autoantibodies appeared but only at a later stage, while the remaining patients remained persistently negative.

All patients, in fasting condition for at least six hours and with normal blood glucose levels, remained at rest in an environment with low sensory stimulation for 15 min before the administration of the tracer, in accordance with international guidelines [22,23]. After an intravenous injection of 3.7 MBq/kg of ^18^F-FDG, the patients underwent both brain and whole-body scan (WBS) PET/CT imaging in the same session. The brain PET/CT was performed 45 min post-injection and lasted for 15 min. This was immediately followed by WBS acquisition, which took approximately 20 min. A low amperage was used for attenuation correction in CT data. The CT scan for the brain used parameters of 3.75 mm slices, 120 kV, and 45 mAs. The WBS scan used 5 mm slices, 120 kV, and 60 mAs. The acquisition was performed without the administration of an intravenous contrast agent. Following the CT scan, a PET scan was performed, covering the same transverse field of view. The acquisition time for each table position was 2 min and 30 s. The PET image data sets were reconstructed iteratively using the CT data for attenuation correction. The co-registered images were displayed on a workstation (Advantage GE Medical System), which allowed for visualization of the PET and CT images separately or in fusion mode across the axial, coronal, and sagittal planes. Images were displayed using a standardized typical gray color scale, with a continuous progression of colors from low to high uptake, according to EANM guidelines [22]. The standardized image display, including upper/lower color scale thresholding, is advocated to ensure appropriate and best-possible interpretable representation of the reconstructed dataset. During the post-processing phase, PET brain images were combined with MRI scans. Areas of reduced or increased tracer uptake, indicating hypo- or hypermetabolism, were considered pathological. Four experienced nuclear medicine specialists (SN, AS, AM, and GM), all with extensive backgrounds in neurologic and oncologic PET imaging, independently interpreted the images. They were informed about the potentially pathological conditions of the patients but were blind to the results of other neurological examinations.

## 3. Results

Table 3 indicates that in the three patients with MRI data supporting the clinical suspicion of AE, specifically cases A, B, and D, the brain PET/CT revealed a decrease in cerebral metabolism.

In case B, the tracer uptake displayed a widespread decline. However, in two cases (A and B), areas of hypermetabolism were evident in specific regions. The CSF analyses consistently yielded negative results during both the initial and follow-up checks for cases A and D. In contrast, case B later tested positive for the presence of anti-Ma2 antibodies, as indicated in Table 2. In this patient, the WBS PET/CT revealed two suspected hypermetabolic foci in the left lung and in perihilar lymph node on the same side, which were not visible at diagnostic CT scan. During surgery, lung adenocarcinoma was confirmed, along with lymph node metastases. The WBS results for cases A and D were negative.

In patient C, the MRI results were ambiguous, making it challenging to differentiate between an ischemic lesion in the subacute phase, glial neoplasia, or encephalitis. However, a brain PET/CT scan revealed a large cortical area in the right hemisphere with absent metabolic activity, which aligned with the MRI results. Additionally, there was reduced tracer uptake in some regions of the right hemisphere. WBS results were negative, and CSF analysis remained persistently negative.

In two cases (E and F) where MRI only identified chronic cerebrovascular disease, brain PET/CT revealed different patterns of cortical uptake abnormalities. In case E, cortical hypermetabolism was predominantly on the left side. In case F, a brain PET/CT scan revealed hypometabolism in some areas of the left hemisphere, along with hypermetabolism in the right hemisphere. Notably, none of these lesions observed on the PET/CT scan were visible on MRI for both cases. The WBS results were negative in both cases. The search for specific autoantibodies in the CSF was consistently negative in both the initial and all follow-up tests.

In patients G, H, I, and M, whose MRI results were inconclusive, brain PET/CT scans revealed different findings. Patient G showed an area of increased FDG uptake in the cerebellar vermis, while patient H exhibited diffuse hypometabolism. In contrast, patient I displayed normal FDG cortical uptake. Patient M had increased metabolism in the posterior cingulate gyrus, bilaterally in the precuneus, and in the cerebellar vermis.

The WBS results were negative for all cases except for patient H, who exhibited increased FDG uptake in the apical region of the right lung’s superior lobe, indicating a pneumonia process. Additionally, the CSF test for specific autoantibodies showed the late presence of anti-GAD65 antibodies in patient G, while the results remained negative for patients H, I, and M.

Patient L, who later tested positive for anti-LGI1 antibodies in CSF tests and could not undergo an MRI due to being a cardiac pacemaker wearer, had PET/CT findings reveal areas of hypermetabolism in several regions. WBS was negative.

Globally, as illustrated in Table 3, 9/11 patients were clinical classified as affected by AE, also based on the results obtained from brain PET/CT. In particular, three of these patients were confirmed to have AE based on CSF tests positive for autoantibodies (cases B, G, and L). Six patients had persistently negative test results (cases A, D, E, F, H, and M) but with clinical symptoms suggestive of AE. Moreover, one patient was classified as having cerebrovascular disease due to ischemia (case C), while the remaining one patient (case I) was classified as experiencing a transient ischemic attack (TIA).

All nine patients with AE received first-line standard systemic treatment consisting of intravenous methylprednisolone, immunoglobulin, and plasmapheresis in four cases. If the initial treatment was unsuccessful or only partially effective, they were given second-line therapy involving the monoclonal antibody rituximab or lacosamide.

Among the nine AE patients, three of these in whom autoantibodies were persistently absent in CSF tests (cases A, E, and M) showed a complete improvement in clinical conditions and no recurrence occurred during the follow-up.

Three AE patients with acute symptoms (cases D, F, and H) and persistently negative CSF tests had a poor response to standard treatment, experienced rapid clinical deterioration despite further therapeutic approaches, and inevitably died.

As illustrated in Table 4, the remaining three AE patients (cases B, G, and L), all of whom tested positive for autoantibodies (specifically, anti-Ma2, anti-GAD65, and anti-GLI1), showed partial improvement in their clinical assessments after receiving their first cycle of drugs and rehabilitative treatment.

However, during the follow-up, all three patients experienced a recurrence of acute symptoms related to encephalitis. They underwent a second brain and WBS PET/CT scan; however, patients B and G also received MRI scans, while patient L could not have an MRI due to a pacemaker.

Specifically, patient B, who had surgery for lung adenocarcinoma during the follow-up period after the first cycle of therapy, experienced a relapse of encephalitis with acute clinical symptoms approximately 24 months after the initial episode. The brain PET/CT scan revealed severe diffuse cortical hypometabolism, particularly marked in the midbrain, which was consistent with the MRI results. The WBS scan was negative. Despite ongoing treatment, this patient experienced a rapid decline in their clinical condition, ultimately leading to death.

**Figure 1:**
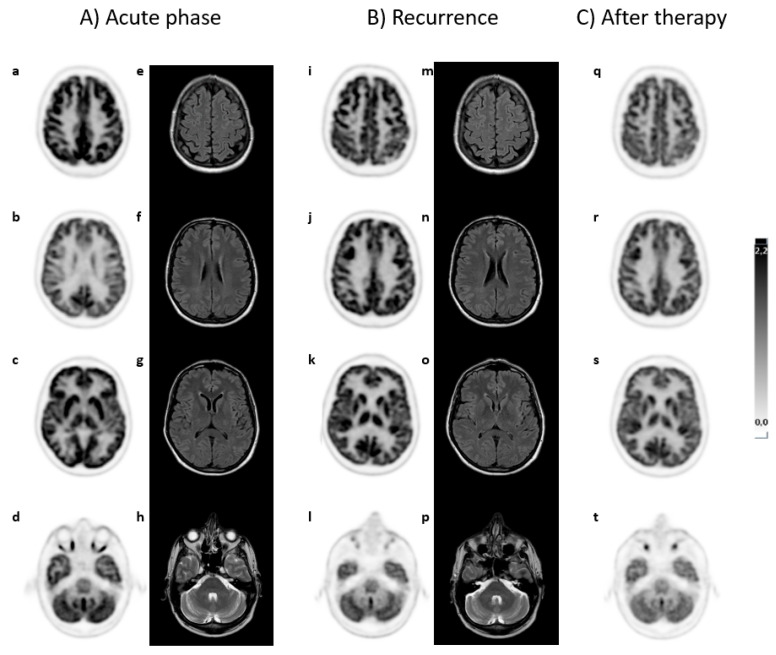
(**A**) A 52-year-old male patient (case G) in the acute phase of autoimmune encephalitis (AE) presented with proteinorrhachia, three IgG bands, and later anti-GAD65 antibodies in cerebrospinal fluid (CSF). A brain ^18^F-FDG PET/CT scan, in transaxial view, indicated increased FDG uptake in the cerebellar vermis (d), while other areas (a–c) exhibited normal uptake. MRI T2/FLAIR (e–h) findings were nonspecific. (**B**) Six months after the initial acute episode, during which the patient was treated with methylprednisolone and immunoglobulin, a recurrence occurred. A repeat brain ^18^F-FDG PET/CT showed new areas of hypermetabolism in the fronto-parietal (i,j) and parieto-temporal (k,l) cortical regions bilaterally, as well as persistent hypermetabolism in the cerebellar vermis (m). The MRI results remained nonspecific (n–p). (**C**) After a second therapy cycle, a new brain ^18^F-FDG PET/CT scan indicated no significant change in the hypermetabolic cortical areas (q–s), while there was a modest reduction in FDG uptake in the cerebellar vermis (t). No MRI was performed at this time. The patient is still alive and remains under observation.

Patient G relapsed six months after the first episode but displayed partial improvement during treatment. A brain PET/CT scan revealed additional hypermetabolic areas in the fronto-parietal and temporal-parietal cortical regions bilaterally, while increased metabolism in the cerebellar vermis persisted. The MRI data remained inconclusive. The patient underwent further cycles of therapy, resulting in partial regression of clinical symptoms. A third brain PET/CT scan indicated that the area of hypermetabolism in the cortical regions continued to persist, although there was a slight reduction in FDG uptake in the cerebellar vermis. The patient is still being monitored. Brain PET/CT and MRI scan for this patient are illustrated in Figure 1.

Patient L, who has a pacemaker, initially showed partial improvement after the first treatment but experienced a recurrence with acute symptoms seven months after the first episode. A brain PET/CT scan showed an increase in FDG uptake in the same areas identified in the baseline PET/CT scan. As a result, additional treatments were introduced, starting with rituximab and later switching to lacosamide due to an allergic reaction to the former. This led to an improvement in the patient’s clinical symptoms, though there were no significant changes in the hypermetabolic brain areas observed on PET/CT. The WBS scan remained negative, and the patient is still alive and under observation.

Finally, the two patients (cases C and I) identified with cerebrovascular ischemia received antiplatelet, antihypertensive, and lipid-lowering treatments, along with antiepileptic medications for one patient experiencing seizures, resulting in improved clinical symptoms.

## 4. Discussion

AE is a neurological condition mediated by autoimmune antibodies and can be linked to paraneoplastic syndromes. Diagnosing AE is particularly challenging because the clinical symptoms can vary greatly and are often nonspecific in the early stages. Additionally, the rapid deterioration of the patient’s condition necessitates prompt treatment, even before specific antibodies are detected in the CSF. Testing for these antibodies can take a long time, and results may occasionally be negative, even after multiple tests.

The criteria for diagnosing AE include clinical symptoms, analysis of CSF for the search of autoantibodies and of serum for different markers, EEG, and MRI [6]. While brain PET/CT is not typically included in the diagnostic approach, some authors have suggested that this procedure may be useful in the diagnostic process for AE [24,25,26,27].

In this study, the effectiveness of PET/CT was retrospectively examined, along with brain metabolic assessment, in the early diagnosis of AE. The analysis involved eleven patients who presented with acute or subacute clinical symptoms suspected to be indicative of AE. These patients also underwent various diagnostic tests, including EEG, MRI, and serum and CSF examinations.

The present data suggest that PET/CT is a useful complementary diagnostic tool for confirming suspected AE in the early stages, in conjunction with other procedures and in line with findings from other authors [24,25,26,27].

Recent data in the literature have indicated that PET/CT outperforms MRI in the early diagnosis of AE. Specifically, 56.5% of AE patients with abnormal PET/CT results had negative MRI findings, highlighting the low sensitivity of MRI [19]. Additionally, other studies [28] report that the overall sensitivity of FDG PET is significantly higher than that of MRI, with rates of 86% compared to 42%. These findings suggest that PET/CT can be a valuable tool in supporting the diagnosis of AE.

The data from the present study, despite being based on a small number of cases in the early phase of the disease, indicated that brain PET/CT scans showed abnormal cortical metabolism in 9/11 (81.8%) of cases classified as AE. The only two exceptions were a case affected by TIA, which exhibited normal uptake on PET/CT, and another case with cerebrovascular ischemia that appeared as hypometabolic area on PET/CT but had clinical and MRI signs suggestive of AE. MRI scans revealed hyperintense T2/FLAIR foci typical of AE, along with vascular or ischemic lesions, in 54.6% of cases. In contrast, the remaining 45.4% were either nonspecific or not feasible for evaluation, which included the patient who experienced the TIA. However, among the nine cases ultimately diagnosed with AE, PET/CT was positive in all instances. In comparison, MRI was suggestive of AE in only three cases (33.3%), while the other five cases in which the exam was performed were classified as either ischemic lesions or nonspecific findings. In only one patient, MRI was not feasible for evaluation due to the presence of a pacemaker.

It was notable that in one instance (case C), PET/CT more accurately defined the vascular nature of a cerebral lesion, while MRI struggled to differentiate between an ischemic lesion in the subacute phase, glial neoplasia, or encephalitis. Recent studies in the literature have also highlighted MRI’s challenge in distinguishing cerebrovascular ischemia from autoimmune inflammatory processes, particularly in the early stages [29].

The increased percentage of metabolic abnormalities on a brain PET/CT compared to MRI findings may be due to functional impairment of neuronal activity rather than structural changes [30].

Furthermore, PET/CT demonstrated its value in evaluating both autoantibody-positive and autoantibody-negative diagnoses of AE, facilitating earlier therapy. In the present study, PET/CT revealed abnormal results in all AE cases, including 66.7% of instances that tested negative for autoantibodies in the CSF. These findings confirm the high sensitivity of PET/CT, even in cases where the search for autoantibodies in CSF has proven ineffective using current assay methods [28].

According to various authors, the absence of autoantibodies in CSF tests is not unusual and does not influence the therapeutic procedures that should be followed. In fact, the response to immunotherapy in these patients suggests that a diagnosis of presumed AE can still be confirmed, even in cases where CSF tests are negative [7,8,9].

Several authors have reported that some autoantibodies, particularly those targeting intracellular proteins like Ma2, may also represent manifestations of paraneoplastic syndromes [30,31,32]. In case B of this study, the combined use of brain and WBS PET/CT scans was effective in identifying not only the encephalitis areas but also other lesions in the lungs, which in this case were classified as an adenocarcinoma nodule with lymph node metastasis, which was confirmed through surgery. Moreover, an inflammatory lung process on WBS was ascertained in another case (case M). Additionally, PET/CT has proven to be beneficial in the follow-up of patients with AE, as it was able to detect recurrences of the disease in three monitored cases after a period of improvement of diseases symptoms. In these patients, PET/CT, which assesses the metabolic state of the brain tissue, was the most effective method for evaluating clinical conditions in response to treatments. MRI was only useful in one case. This finding supports the value of PET/CT in monitoring AE-affected patients, a perspective also endorsed by other researchers [33].

The limitations of this study should be highlighted.

Firstly, it is important to note that the study is retrospective and conducted at a single center, which resulted in a small sample size of patients. Additionally, some patient data may be missing or not recorded in the medical records. This limited number of cases prevents drawing more definitive conclusions, as a larger patient cohort is needed for statistical evaluation. Therefore, this preliminary study would benefit from a larger series of cases, ideally through multicenter collaboration.

Furthermore, the limited number of cases in which the CSF was mostly negative for specific autoantibodies prevents defining specific patterns in PET/CT scans. The metabolic abnormalities observed in this series varied, ranging from a general reduction in cerebral metabolism to specific areas showing either increased or decreased tracer uptake.

In this study, the qualitative analysis of PET/CT could be enhanced through quantitative analysis, which would provide more valuable insights into the metabolic abnormalities present in patients with AE.

## 5. Conclusions

The current data, although based on a limited number of patients, suggest that brain ^18^F-FDG PET/CT scans may serve as a valuable complementary diagnostic tool to standard procedures for the early identification of suspected AE, especially when used alongside other tests. If used early, this method could help prevent delays in initiating immunotherapy or other treatments aimed at improving clinical outcomes.

In this series of patients, PET/CT scans demonstrated greater sensitivity than MRI because the early phase of AE predominantly presents with brain phenomena that reflect functional and inflammatory processes, which occur before the structural changes typically identified by MRI.

The use of PET/CT becomes especially crucial when MRI results are unclear or when MRI is not feasible, particularly if CSF tests are inconclusive and remain negative in follow-up evaluations. Moreover, negative PET/CT results can help rule out the disease. Additionally, PET/CT has proven valuable for monitoring patients, assessing their response to treatment, and detecting any recurrence of the disease. Finally, the combination of WBS and brain PET/CT has shown utility in identifying extracerebral neoplastic and inflammatory lesions, although this association should not be considered for routine use. However, the results of this study need to be confirmed with a larger sample size.

## Figures and Tables

**Table 1:** Demographic characteristics and clinical symptoms of eleven patients suspected of AE in the early stage.

Cases	Sex	Age	Clinical Symptoms											
			Headache	Fever	Dizziness	Drowsiness	Reduced/Loss of Consciousness/Confusion	Cognitive Impairment	Behavior Disturbance	Seizures	Motor Disorders/Tremors	Speech Alterations	Hallucinations	Ocular Disorders
A	M	74	+				+		+		+		+	
B	F	72	+		+	+	+				+			+
C	M	57								+				
D	F	22	+	+			+	+			+			
E	M	76	+				+	+			+			
F	M	60		+			+	+			+			+
G	M	52	+						+		+	+		
H	M	79	+		+	+	+							
I	M	67		+			+	+			+	+		
L	M	71	+				+		+	+	+			
M	F	69	+				+	+	+		+	+	+	

**Table 2:** Diagnostic procedures performed in the eleven cases with suspected AE in the early stage.

Cases	EEG	MRI (Hyperintensity in T2/FLAIR)	SERUM (for Research of Tumor Markers, Bacterial/Viral Agents for Meningitis, and Infectious Encephalitis)	CSF (for Research of Autoantibodies)
A	Nonspecific sharp and slow theta-delta waves in F regions.	P and O lobes bilaterally, mostly on the left (suspected AE).	Negative.	Prot. and three IgG bands. Persistently negative for Ab.
B	Not performed due to poor patient cooperation.	Am bilaterally, mostly on the right. Left Th (suspected AE).	Negative.	Late appearance of Ab anti-Ma2.
C	Right anterior T epileptogenic focus.	Right T region, insula, Hip, and para-Hip (doubtful of ischemic lesion in subacute phase/glial neoplasia/encephalitis).	Negative.	Persistently negative for Ab.
D	Nonspecific.	F, T, Hip, and insula bilaterally, mostly on the left (suspected AE).	Negative.	Persistently negative for Ab.
E	Nonspecific.	Posterior P, F and basal nuclei (mostly Cau and Len) bilaterally (suspect ischemic lesions).	Negative.	Persistently negative for Ab.
F	Nonspecific.	P-T bilaterally and left corona radiate (ischemic lesions).	Negative.	Prot. Persistently negative for Ab.
G	Nonspecific.	Nonspecific.	Negative.	Prot. and three IgG bands. Late appearance of Ab anti-GAD65.
H	Not performed due to poor patient cooperation.	Nonspecific.	Negative.	Prot. Persistently negative for Ab.
I	Nonspecific.	Nonspecific.	Negative.	Prot. Persistently negative for Ab.
L	Theta-delta activity in right F-T areas.	Not performed (PW).	Negative.	Late appearance of Ab anti-LGI1.
M	Normal.	Nonspecific.	Negative.	Prot./Pleoc. Persistently negative for Ab.

AE: autoimmune encephalitis; EEG: electroencephalogram; MRI: magnetic resonance imaging; CSF: cerebrospinal fluid; F: frontal; T: temporal; P: parietal; O: occipital; Am: amygdala; Th: thalamus; Hip: hippocampus; Cau: caudate; Len: lenticular; PW: pacemaker wearer; Prot: proteinorrachia; Ab: autoantibody; Pleoc: pleocytosis.

**Table 3:** Brain and WBS ^18^F-FDG PET/CT in the eleven patients suspected of AE in the early stage.

Cases	Brain ^18^F-FDG PET/CT			WBS ^18^F-FDG PET/CT	Definitive Clinical Classification
	Decreased Metabolism	Increased Metabolism	Normal Metabolism		
A	Reduced FDG uptake in lateral prefrontal, inferior P, lateral O, lateral T, anterior and posterior CG, and precuneus bilaterally.	Increased FDG uptake in left F and TM regions.		Negative.	AE
B	FDG diffuse global reduction.	Focal areas of increased FDG uptake in left superior P and T-P, and right T regions.		Two hypermetabolic areas were identified in the left lung. One was located in a calcified nodule in the dorsal segment of the upper lobe, while the other was found in the perihilar lymph node on the same side as the tumor, which was identified as adenocarcinoma with metastasis, discovered during surgery.	AE + lung metastatic carcinoma.
C	FDG uptake absent in T region, insula, Hip and para-Hip and reduced in P and lateral O areas and Th in the right hemisphere.			Negative.	Cerebrovascular ischemia.
D	Reduced FDG uptake in prefrontal lateral, SM, and posterior CG of left hemisphere. O lateral and visual regions bilaterally.			Negative.	AE
E		Increased FDG uptake in superior PG, cuneus, precuneus, superior TG, lingula, and inferior OG bilaterally.		Negative.	AE
F	Reduced FDG uptake in F, anterior CG, and TM regions of the left hemisphere.	Increased FDG uptake in right superior P and T lateral regions.		Negative.	AE
G		Increased FDG uptake in CV.		Negative.	AE
H	Global decrease in FDG uptake.			Increased FDG uptake in the apical area of the right lung consistent with an inflammatory process.	AE + inflammatory lung process.
I			Generally normal reduction in FDG uptake.	Negative.	TIA
L		Increased FDG uptake in postcentral gyrus, precentral gyrus, superior P lobule, angular gyrus, and precuneus bilaterally mostly on the left hemisphere.		Negative.	AE
M		Increased FDG uptake in posterior CG and precuneus regions bilaterally, and CV.		Negative.	AE

AE: autoimmune encephalitis; WBS: whole-body scan; ^18^F-FDG PET/CT: ^18^Fluoro-fluorodeoxyglucose positron emission tomography/computed tomography.; F: frontal; T: temporal; P: parietal; O: occipital; CG: cingulate gyrus; Th: thalamus; Hip: hippocampus; SM: sensorimotor; TM: temporo-mesial; PG: parietal gyrus; TG: temporal gyrus; OG: occipital gyrus; CV: cerebellar vermis; TIA: transient ischemic attack.

**Table 4:** Clinical data, MRI, and ^18^F-FDG PET/CT in the follow-up of three AE cases with partial response to treatment.

Cases	CSF Autoantibodies	First Cycle of Treatment	Response to Treatment	AE Recurrence			Second Cycle of Treatment	Response to Treatment		
		Type of Therapy		Clinical Symptoms	MRI	Brain PET/CT	Type of Therapy	Clinical Symptoms	MRI	Brain PET/CT
B	Ab anti-Ma2	Mth Ig Plasm	Slight improvement in Clin-Symp	Twenty-four mts after the first acute episode: progressive appearance of OM, BD and MD.	Hyperintensity in T2/FLAIR in ponto-mesencephalic and TM regions bilaterally accompanied by severe atrophy.	More diffuse global hypometabolism with focal areas of hypermetabolism in the left superior P and T-P regions, as well as right T regions, showing marked FDG uptake in the midbrain.	MthPlasm Rit	No response to treatment with rapid loss of consciousness and subsequently death.	Not performed due to patient death.	Not performed due to patient death.
G	Ab anti-GAD65	Mth Ig	Slight improvement in Clin-Symp.	Six mts after the first acute episode: appearance of further MI, SP-D, and BD.	No evidence of altered signal in the sub- and supra-tentorial parenchymal structures.	Appearance of new areas of hypermetabolism in and T-P cortical regions bilaterally, and persistence in CV.	Mth Ig	Partial improvement of Clin-Symp (patient still under observation).	Not performed.	No significant change in hypermetabolism observed in the FP and TP cortical regions bilaterally, along with a modest reduction in the CV.
L	Ab anti-LGI1	AED Mth	Slight improvement in Clin-Symp.	Seven mts after the first acute episode: appearance of confusion, drowsiness, dizziness, loss of consciousness, and pilomotor seizures.	Not performed (PW).	More pronounced hypermetabolism in the postcentral gyrus, precentral gyrus, superior P lobule, angular gyrus, and precuneus bilaterally, predominantly on the left side.	AED Mth Plasm Rit (replaced by Lac for allergy episodes)	Partial improvement of Clin-Sympt (patient remains under observation).	Not performed (PW).	No significant change of hypermetabolism in the regions observed on the pre-therapeutic brain PET/CT scan.

AE: autoimmune encephalitis; CSF: cerebrospinal fluid; MRI: magnetic resonance imaging; ^18^F-FDG PET/CT: ^18^Fluoro-fluorodeoxyglucose positron emission tomography/computed tomography; Mth: methylprednisolone; Ig: immunoglobulin; Plasm: plasmapheresis; AED: Anti-epileptic drugs; Clin-Symp: clinical symptoms; mts: months; OM: ocular-motor; BD: behavior disturbance; MD: movement disorders; MI: motor impairment; SP-D: speech disorders; TM: temporomesial; PW: pacemaker wearer; P: parietal; T: temporal; CV: cerebellar vermis; Rit: rituximab; Lac: lacosamide.

## Data Availability

The original contributions presented in this study are included in the article. Further inquiries can be directed to the corresponding author(s).
